# Host-Specialist Dominated Ectomycorrhizal Communities of *Pinus cembra* are not Affected by Temperature Manipulation

**DOI:** 10.3390/jof1010055

**Published:** 2015-04-30

**Authors:** Georg Rainer, Regina Kuhnert, Mara Unterholzer, Philipp Dresch, Andreas Gruber, Ursula Peintner

**Affiliations:** 1 Institute of Microbiology, University of Innsbruck, Technikerstraße 25d, A-6020 Innsbruck, Austria; E-Mails: georg.rainer@student.uibk.ac.at (G.R.); regina.kuhnert@uibk.ac.at (R.K.); mara.unterholzer@student.uibk.ac.at (M.U.); philipp.dresch@uibk.ac.at (P.D.); 2 Institute of Botany, University of Innsbruck, Sternwartestraße 15, A-6020 Innsbruck, Austria; E-Mail: a.gruber@uibk.ac.at

**Keywords:** keystone mycobionts, *Suillus placidus*, afforestation, sustainable mycorrhiza inoculation

## Abstract

Ectomycorrhizae (EM) are important for the survival of seedlings and trees, but how they will react to global warming or changes in soil fertility is still in question. We tested the effect of soil temperature manipulation and nitrogen fertilization on EM communities in a high-altitude *Pinus cembra* afforestation. The trees had been inoculated in the 1960s in a nursery with a mixture of *Suillus placidus*, *S. plorans* and *S. sibircus.* Sampling was performed during the third year of temperature manipulation in June and October 2013. Root tips were counted, sorted into morphotypes, and sequenced. Fungal biomass was measured as ergosterol and hyphal length. The EM potential of the soil was assessed with internal transcribed spacers (ITS) clone libraries from in-growth mesh bags (MB). Temperature manipulation of ± 1 °C had no effect on the EM community. A total of 33 operational taxonomic units (OTUs) were identified, 20 from the roots, 13 from MB. The inoculated *Suillus* spp. colonized 82% of the root tips, thus demonstrating that the inoculation was sustainable. Nitrogen fertilization had no impact on the EM community, but promoted depletion in soil organic matter, and caused a reduction in soil fungal biomass.

## 1. Introduction

The Swiss pine (*Pinus cembra*) is typical tree species for subalpine to alpine habitats with an altitudinal range from 1500 to 2500 m above sea level (a.s.l.) [1]. It is well adapted to high altitude ecosystems with harsh environmental conditions, like low winter temperatures, elevated ozone levels and radiation, and thus is an important tree species of the Alpine timberline ecotone [2,3]. The growth of *P. cembra* timberline stands is limited by mean daily air temperatures below 6–8 °C during the vegetation period [4,5]. Thus, global warming might enable an invasion of higher lying areas by *P. cembra*. The bird *Nucifraga caryocatates* might play an important role in the upward spread of *P. cembra*, as it acts as a seed dispersal vector [6].

*Pinus cembra* trees are usually strongly mycorrhized [7,8]. Three basidiomycete species of the genus *Suillus* form a strictly host-specific symbiosis with these trees: *S. plorans*, *S. placidus* and *S. sibiricus*. The advantage of host-specialism lies in the highly efficient nutrient and water transfer between the symbiosis partners, and in the exclusion of mycoheterotrophy [9]. Therefore, host-specialists are often the dominating ectomycorrhizal species in high-altitude areas with extreme conditions [10,11]. Besides these host-specialist *Suillus* spp., there are a number of other generalist ectomycorrhizae (EM) fungi that are associated with *P. cembra*, e.g., *Amphinema byssoides*, *Thelephora terrestris* or *Cortinarius* spp., but they are usually occurring in low abundance only [8,12]. The function of EM symbiosis ranges from trophic interactions to protective effects [13]. For a better estimation of future scenarios and for a proactive planning for the conservation of *P. cembra* habitats, it is therefore very important to gain knowledge on the influence of climate change on the EM symbiosis of these plants.

The current distribution of *P. cembra* has been strongly shaped by historical anthropogenic influences. The *P. cembra* areal has been significantly restricted in past centuries by clearing and pasturing [14,15,16,17]. Due to continuous grazing, the slopes of the Sellrain Valley remained deforested for centuries [18]. Only after serious avalanche disasters in the winters of 1950 and 1951, a large-scale afforestation was started in Haggen (municipality St. Sigmund im Sellrain), with the aim to establish a protective forest above threatened settlements [18]. However, the first attempts in 1963 failed when all newly introduced Swiss pine seedlings died off, most likely caused by the absence of suitable mycorrhiza partners after the hundreds of years of deforestation [19]. Finally, a long-term afforestation could be successfully established in 1964 after artificial mycorrhization of seedlings with several *P. cembra* host-specialists of the genus *Suillus*. The afforestation was continuously enlarged until 1986, and was so successful that the first planted tree populations had to be thinned out after 34 years [20]. This proves that suitable EM partners are crucial for the success of *P. cembra* afforestations and that they can be successfully introduced as inoculated seedlings. Swiss pine trees associated with suitable mycobionts have a higher vitality and produce more biomass than trees without [19,21,22].

For future management practices, it is very important to know if the EM inoculation carried out in the 60s of the last century is sustainable or not: the mycobiont species composition can change during the tree development, and seedlings may have a different species composition than adult trees [8,23,24]. First investigations performed on up to 34-year-old *P. cembra* trees in Haggen in 1997 indicated that the inoculated host-specialist *Suillus* spp. were still the dominating mycobionts on all age categories of *P. cembra* in this area: only small differences were found in the species composition of young and adult trees [20]. However, this investigation was performed with classical morphological methods, thus not allowing for discrimination between the three mycorrhiza-nodule-forming *Suillus* specialists. Other inventories of the fungal EM partners of *P. cembra* have been based on co-occurring fruiting bodies only [25], however, below-ground diversity of fungal communities is not entirely displayed by fruiting bodies above ground [26]. This study is the first, which thoroughly investigates the EM associations of *P. cembra* in a high-altitude afforestation with molecular methods. We primarily wanted to answer the question, whether inoculation with mycorrhizal specialist species can be sustainable over more than 30 years, and if these EM communities are affected by N fertilization. All actual climate models predict a warming in the Alps, although they do not agree on the dimension of temperature change [27]. Therefore, an additional aim of this study was to investigate the effect of changed soil temperatures based on soil temperature manipulations. We also considered the mycorrhizal inoculum present in the soil based on rDNA ITS clone libraries of in-growth mesh bags (MBs) [28,29].

## 2. Material and Methods

### 2.1. Site Descriptions

The study site is located in the Sellrain Valley (Austrian Central Alps) on a high altitude *P. cembra* afforestation on the Haggener Sonnberg (1720–2030 m a.s.l.). Sampling was performed in a population of about 25–30-year-old Swiss pine (*Pinus cembra*) of 3–4 m tree height and about 25% canopy coverage at an altitude of 2026 m a.s.l. with SSW exposition and an inclination of 35–40° [20]. The soil is classified as a haplic podzol, which is derived from mica slate and gneiss. The plant community at this site is considered *Vaccinio-Pinetum cembrae* with high abundances of *Calluna vulgaris*, *Vaccinium vitis-idea*, *V. myrtillus*, *Luzula silvatica*, *Nardus stricta*, *Deschampsia flexuosa* and *Juniperus communis*. A detailed description of geology, soil properties, and vegetation was given by Neuwinger [18]. The sampling area is typically free of snow between April or May and October or November. The mean air temperature in the sampling area was 7.5 °C during the vegetation period (15 April to 28 October 2013). The highest air temperature of 18.4 °C was measured on 28 July 2013. The precipitation during the vegetation period was 890 mm.

### 2.2. Experimental Design

Ten separate plots of about 10 m^2^, which all contained three *P. cembra* trees, were randomly defined within the sampling area. Four of these plots were used for temperature manipulation: in two plots, radial soil warming was inhibited by shading of the rooting zone using non-transparent foils to generate reduced soil temperatures (K), while heat-trapping under transparent glasshouse foils was used for artificial soil warming (W) in two other plots. The respective foils were fixed on frames mounted about 20 cm above the ground. The sides were left open to allow for air circulation. Two plots were used as control (CO). These soil temperature manipulations were carried out from 2011–2013 during the growing season. Two additional plots were defined for a nitrogen fertilization experiment and were fertilized with nitrogen (N) following recommendations of Kilian and Mueller [30]. The N plots were fertilized twice, in spring 2012 and 2013, with calcium ammonium nitrate (NAC 27 N, Borealis L.A.T, AT). This nitrogen fertilizer contains 27% nitrogen, 13.5% in the form of NO_3_-N and 13.5% in the form of NH_4_-N and was applied at the beginning of the vegetation period in 2012 and 2013 at a nitrogen concentration of 10 g·m^−2^. The natural vegetation was mowed in all manipulated plots (K, W, N) and in two control plots (CO) without temperature or fertilizer manipulation. Two plots with natural vegetation were used as additional control (CM).

### 2.3. Soil Parameters

Soil temperature (T 107 Temperature Probe, Campbell Scientific, Shepshed, UK) and volumetric soil water content (EC 5 Soil Moisture Sensor, Decagon Devices Inc., Pullman, WA, USA) were continuously measured at about 10 cm soil depth using four sensors per treatment (W, K, CO). Data were recorded with a data logger (CR1000, Campbell Scientific, UK) programmed to record 30-min averages.

Soil sampling was performed after snow melt on 8 May 2013, and at the end of the vegetation period on 28 October 2013. In order to determine soil parameters and ergosterol measurements, six random samples were taken from the Ah-horizon of each plot: after removing the organic layer, a spade was used to dig to a depth of about 10 cm. Soil samples were sieved with a 2 mm mesh, and stored at −20 °C.

The determination of soil water content (SWC), pH and soil organic matter (SOM) occurred according to Schlichtling, *et al.* [31]. Briefly, the soil dry weight was determined by drying soil samples at 105 °C to constant weight. The percentage of soil water content (SWC) was calculated as fresh weight minus dry weight of soil samples.

The measurement of pH was carried out potentiometric in a CaCl_2_ solution (0.01 mol·L^−1^). Soil organic matter was determined from the weight loss following ignition in a muffle furnace (CWF 1100, Carbolite, Neuhausen, Germany) at 430 °C to equilibrium constant. Total N and total C in the samples were measured using a CHN analyzer (Tru-Spec^®^, Leco Corp., St. Joseph, MI, USA).

### 2.4. Soil Fungal Biomass

Soil fungal biomass was determined based on ergosterol content. Frozen soil samples (−20 °C) were thawed at 4 °C before analysis. The measurement was conducted as described by Spath *et al.* (2014) [32].

### 2.5. In-Growth Mesh Bags

In-growth MBs (size 4 cm^2^) were constructed of a polyester mesh (53 μm mesh size, neoLab, Heidelberg, Germany) by sewing the edges together and filled with 15 g sterile, acid-washed quartz sand (0.36–2.00 mm grain size, Dehner, Rain, Germany) according to Wallander, Nilsson, Hagerberg and Baath [29].

Three MBs per plot resulting in a total of 30 MBs were buried on 6 June 2013 and harvested on 28 October 2013 (144 days). Mesh bags were transported to the laboratory and immediately opened at one edge with sterile scissors. Then, the quartz sand was filled into several sterile 2 mL microcentrifuge tubes. These tubes were either frozen at −80 °C until being processed, or the DNA-extraction was performed directly. Mycelial strands and rhizomorphs adhering to the inner surface of the MBs were stored separately in sterile microcentrifuge tubes for further evaluations.

### 2.6. Hyphal Length in Mesh Bags

Comparison of the hyphal length in MBs allowed surveying the extent of fungal in-growth during the vegetation period (time of burial) in the different plots. Determination of hyphal length was carried out based on a fluorescence stain technique with Calcofluor White M2R [33]. A test tube was filled with 0.5 g of quartz sand and with 2 mL phosphate buffer (60 mmol·L^−1^, pH 7.5), mixed and shaken for 10 min. Test tubes were put in an ultrasonic cleaner for up to one minute to detach residual hyphae from quartz sand grains. One milliliter Calcofluor White (15 μg·mL^−^^1^) was added and stained for 3 min. To remove excess dye, the stained suspension was filtered through a 0.4 μm pore size filter (Millipore HTBP02500 Isopore Black, Merck Millipore, Darmstadt, Germany) fixed upon a 25 mm diameter filter holder (Swinnex, Merck Millipore, Germany) and washed out with distilled water. Filters were put on a microscope slide, air-dried, cleared in immersion oil and finally covered with a cover glass. All samples were analysed with a fluorescence microscope (Microphot-SA, Nikon, Tokyo, Japan) with a high intensity mercury light source and a UV-2A fluorescence filter (Nikon, Japan). From each filter, 10 sections were randomly selected at a magnification of 200 fold and photographed with an 8-Bit-Digicam (Digital SightDS-U1, Nikon, Japan). Dyed hyphae were measured digitally using the software program NIS-Elements Documentation (ver. 3.00, Nikon, Japan).

### 2.7. Sampling and Processing of Ectomycorrhizae

For sampling of EM, a soil-corer with a diameter of 4 cm was used (approximate soil volume: 250 mL). Five soil samples were taken from each plot. The samples were immediately transported to the laboratory where they were stored at 4 °C for further analysis. All samples were processed within 5 days. Mycorrhiza sampling was performed in June at the start of the growing season and in October at the end of the growing season. The whole root systems sampled in each soil core was gently washed in tap water using a 2 mm sieve to remove most of the soil and organic debris, while minimizing damage to the roots. Organic material adhered tightly to the root system was removed with forceps.

Living roots were identified on the basis of a turgid appearance and possessing white cortical cells [34]. All mycorrhized and non-mycorrhized root tips were analysed and were counted at 10–100× magnification using a stereomicroscope (SMZ800 Zoom, Nikon, Japan). Furthermore, photos were taken with NIS-Elements (Nikon, Japan). Ectomycorrhizal morphotypes (MTs) were defined based on characteristics of the fungal mantle, such as colour, emanating elements, mantle layer, and hyphal strands [35,36]. From each sample, at least one small piece of mantle of each MT was stored in PCR strips containing 20 µL of PCR-grade water at −80 °C until further processing. All other root tips were dried for determination of dry weight at 105 °C to constant weight.

### 2.8. Molecular Identification of Ectomycorrhiza

Molecular identification of the various MTs was carried out based on a modified protocol of the Rapido-PCR [37]. A volume of 30 µL PCR reaction mix was added to every EM sample in 20 µL PCR-grade water. The primer pair ITS1F and ITS4 [38,39] was used for amplification. The PCR mixture contained the following reagents (final concentration): BSA (200 µg·mL^−1^), 1× Buffer S (1.5 mmol·L^−1^ MgCl_2_, 10 mmol·L^−1^ Tris, 50 mmol·L^−1^ KCl, dNTPs (0.2 µmol·L^−1^), peqGOLD Taq DNA polymerase (0.75 units) (all Peqlab, Erlangen, Germany) and of the two primers (400 µmol·L^−1^, each; Microsynth, Balgach, Switzerland). Amplification was performed in a thermal cycler (Primus 96, Peqlab, Germany) with a reaction volume of 50 μL. The following thermocycling pattern was used: an initial denaturation at 94 °C for 3 min; followed by 35 cycles of 94 °C for 30 s, 50 °C for 30 s and 72 °C for 45 s; and a final elongation at 72 °C for 7 min. Negative controls (PCR-grade water) were included in each PCR reaction. PCR products were electrophoresed in 1.5% (*w*/*v*) agarose gel stained with ethidium-bromide.

PCR products of the MTs forming mycorrhizal nodules and coralloid mycorrhizae were analyzed using restriction fragment length polymorphism (RFLP) carried out with the buffer M (GeneCraft, Cologne, Germany), BSA and restriction enzyme BsuRI (GeneCraft, Germany) following the manufacturer’s instructions. The ITS-regions of several representative samples from each RFLP pattern were sequenced using the primer ITS4. The PCR products of all other MTs were directly sequenced.

### 2.9. Ectomycorrhiza Inoculum Potential of the Soil

DNA-extraction and amplification were performed for each MB separately as described by Kuhnert, Oberkofler and Peintner [28]. For each plot, PCR products from three MBs were pooled at equal DNA concentrations. Pooled PCR products were purified using the GenElute™ PCR Clean-Up Kit (Sigma Life Science, St. Louis, MO, USA) and cloned using the TOPO TA Cloning^®^ kit (Invitrogen, Carlsbad, CA, USA) in combination with chemically competent *Escherichia coli* (TOP10 cells, Invitrogen, Carlsbad, CA, USA) following the manufacturer’s instructions. The clone PCR mixture contained the same reagents and concentrations as described above for the EM. Cycling parameters were 94 °C for 10 min, followed by 30 cycles of 94 °C for 1 min, 53 °C for 30 s, 72 °C for 1 min with a final extension of 72 °C for 10 min. Twenty clones per plot were sequenced. Sequencing was performed by Microsynth GmbH (CH).

### 2.10. Sequence Analysis

DNA sequences were controlled and edited using Sequencher^®^ (ver. 5.2.3, Gene Codes, Ann Arbor, MI, USA). Ribosomal DNA ITS sequences were assembled with a minimum sequence identify of 99% and a minimum overlap of 85%, thus representing one operational taxonomic unit with 99% sequence similarity (OTU). BLAST searches were performed using the public databases NCBI [40] and UNITE [41,42].

### 2.11. Data Handling and Statistical Analysis

Data are given as arithmetic means with standard deviations. Statistical analysis was carried out with STATISTICA version 9.1 StatSoft, Inc. (2010, StatSoft, Tulsa, OK, USA). Normal distribution of the data was tested by the Kolmogorow-Smirnoff-goodness-of-fit test and by examination of histograms. To test the effect of the season, significant differences between the means of the measured variables were calculated by the standard *T*-test of independent samples. If the data showed no variance of homogeneity, the *T*-test was replaced by the Mann-Whitney *U*-test. The influence of temperature manipulation and N fertilization between the plots was tested by the Kruskal-Wallis One Way Analysis of Variance on Ranks. The significance level was *p* < 0.05 for all tests. If standard deviations were higher than arithmetic means, no statistical tests were carried out, but data were examined based on box-and whisker plots with median and 95% percentiles. Diversity indices, indicator species testing and multi response permutation procedure were calculated with PC-ORD (ver. 6.0, MjM Software, Gleneden Beach, OR, USA) [43].

## 3. Results

### 3.1. Soil Temperature and Soil Moisture

The soil temperature manipulation worked well: temperatures differed significantly between cooled, warmed and control plots. Temperature differences of ± 1.3 °C, SD = 0.6 were obtained during the vegetation period (mean soil temperature: warmed plots = 10.3 °C, SD = 3.9; cooled plots = 7.7 °C, SD = 3.2; Figure S1). The overall mean soil temperature in 10 cm soil depth was 9.0 °C (SD = 3.2, *n* = 197) during the vegetation period (15 April 2013 to 28 October 2013). The highest soil temperature of 17.3 °C was measured in the warm plots on 3 August 2013.

The relative soil moisture as retrieved from data loggers was not significantly different between plots (Figure S1), and there were no significant differences in the percentage of SWC between plots in October. In May, the SWC was significantly lower in warm plots than in the control plots (CO and CM). However, the SWC differed significantly between May and October (Table 1). In May, it ranged from 44% to 51%, and in October, it ranged from 38% to 47%.

### 3.2. Soil pH

In May, soil pH values were significantly higher in CM and N plots (4.2) than in the plots W, K, CO (pH 3.8–3.9) (Table 1). In October, pH values were not significantly different between plots, nor did they differ significantly between May and October.

### 3.3. Soil Organic Matter

Soil organic matter varied from 22% to 29% in the sampling site, with significantly higher values in May than in October (Table 1). The highest mean SOM values were measured in the control plots with vegetation, with 29% (SD = 5, *n* = 10) in May and 27% (SD = 5, *n* = 10) in October. Significant differences between the plots were only found between nitrogen plots and control plots with vegetation in October.

**Table 1:** Mean values and standard deviations (*n* = 10) of soil organic matter (SOM, %), soil water content (SWC, %) and pH values from the five treatments of *P. cembra* in Haggen in May and October 2013. W = warm, K = cold, CO = control with cut vegetation, CM = untreated control, N = nitrogen fertilization. Results are given as mean (± SD). Significant differences between seasons are given at *p* < 0.05 (^1^ *p* < 0.03; ^2^ *p* < 0.0001). For the effects of temperature manipulation and fertilization, values with the same suffix letter (a, b) are not significantly different at the *p* < 0.05 level.

Measured Variable	W	K	CO	CM	N
*May*					
SOM ^1^	28.1 ± 6.1 ^a^	28.7 ± 6.7 ^a^	27.5 ± 7 ^a^	29.5 ± 4.5 ^a^	26.9 ± 2.2 ^a^
SWC ^2^	43.6 ± 3.2 ^a^	47.0 ± 7.0 ^a, b^	50.5 ± 3.2 ^b^	51.8 ± 2.2 ^b^	48.3 ± 3.7 ^a, b^
pH	3.8 ± 0.1 ^a^	3.9 ± 0.2 ^a^	3.9 ± 0.2 ^a^	4.2 ± 0.2 ^b^	4.2 ± 0.0 ^b^
*October*					
SOM ^1^	26.5 ± 3.4 ^a^	25.5 ± 5.0 ^a^	25.8 ± 4.8 ^a^	27.2 ± 4. 2 ^a^	22.1 ± 1.9 ^a^
SWC ^2^	37.5 ± 5.1 ^a^	37.4 ± 7.3 ^a^	42.3 ± 4.3 ^a, b^	43.9 ± 6.4 ^a, b^	46.6 ± 9.6 ^b^
pH	3.9 ± 0.1 ^a^	4.0 ± 0.1 ^a^	4.0 ± 0.1 ^a^	4.0 ± 0.1 ^a^	4.0 ± 0.0 ^a^

### 3.4. Root Dry Weight

The root dry weight differed significantly between May and October, but within seasons there were no significant differences between the plots. In May, the values varied between 0.3 g·L^−1^ soil (SD = 0.3, *n* = 10) in CM and 1.1 g·L^−1^ soil (SD = 1.8, *n* = 10) in W. In October, the root dry weight ranged between 0.7 g·L^−1^ soil (SD = 0.7, *n* = 10) in the N and 1.2 g·L^−1^ soil (SD = 1.6, *n* = 10) in W plots. The root dry weight increased during the growing season with exception of the plot CM (Table 2).

### 3.5. Soil Fungal Biomass

The soil fungal biomass decreased significantly during the growing season with mean ergosterol values of 47.6 µg·g^−1^ soil (SD = 15.4, *n* = 53) in May, and 36.1 µg·g^−1^ soil (SD = 12.4, *n* = 54) in October. Ergosterol concentrations were not significantly different between the plots in May. However, in October the ergosterol content in the nitrogen plots showed a drastic decrease and was 45% lower than the mean (Table 2).

### 3.6. Hyphal Length for Estimation of Fungal Biomass in Mesh Bags

Fungal in-growth during the vegetation period was reduced in the two manipulated plots (W, N) compared to controls (CO, CM). The highest values were found in CM with 9.9 m·g^−1^ soil (SD = 7.8, *n* = 9) and in CO with 9.7 m·g^−1^ soil (SD = 5.5, *n* = 9) (Table 2).

### 3.7. Ectomycorrhiza Root Tips and Their Degree of Mycorrhization

**Figure 1:**
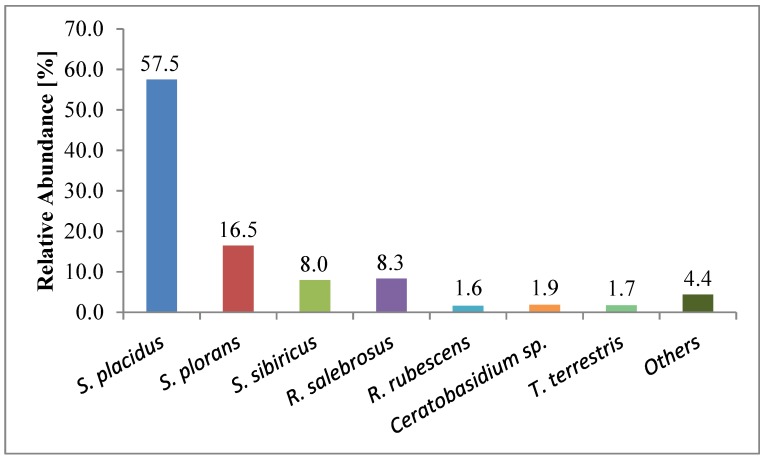
Relative abundances (%) of the most important ectomycorrhizal partner of *P. cembra* in the Haggen afforestation.

We could not detect differences between plots neither for the number of EM root tips·L^−1^ soil, nor for the degree of mycorrhization (Figure 1, Table 2). The number of EM root tips increased during the vegetation period: 1399 EM root tips·L^−1^ soil (SD = 1340, *n* = 50) were detected in May, compared to 2263 EM root tips·L^−1^ soil (SD = 2260, *n* = 50) in October; variation between samples was very high. The degree of mycorrhization was generally very high (99.4%, SD = 1.1%, *n* = 100).

**Table 2:** Mean values and standard deviations of mycorrhized root dry weight (g·dry weight·L^−1^ soil), EM root tips (root tips·L^−1^ soil), mycorrhization (%), ergosterol (µg·g^−1^ soil), and hyphal length (m·g^−1^ soil) as measured in the *P. cembra* treatments in May and October 2013. (Measurement per treatment: *n* = 10; except hyphal length *n* = 9). W = warm, K = cold, CO = control with cut vegetation, CM = untreated control, N = nitrogen fertilization. Results are given as means (± SD). Significant differences between seasons are given at the *p* < 0.05 level (^1^ *p* < 0.01; ^2^ *p* < 0.003; ^3^ *p* < 0.0002). For the effects of temperature manipulation and fertilization, values with the same suffix letter (a, b) are not significantly different at *p* < 0.05.

Measured Variable	W	K	CO	CM	N
*May*					
Root dry weight ^1^	1.1 ± 1.8 ^a^	0.5 ± 0.4 ^a^	1.3 ± 1.6 ^a^	0.3 ± 0.3 ^a^	0.7 ± 0.5 ^a^
EM root tips	1828 ± 1919	1590 ± 1192	1374 ± 1209	797 ± 613	1440 ± 1493
Mycorrhization ^2^	99.8 ± 0.5 ^a^	100 ± 0.1 ^a^	99.4 ± 1.8 ^a^	99.6 ± 0.9 ^a^	99.3 ± 0.9 ^a^
Ergosterol ^3^	51.6 ± 16.3 ^a^	45.8 ± 18.1 ^a^	42.2 ± 11.1 ^a^	51.3 ± 18.3 ^a^	47.4 ± 8.4 ^a^
*October*					
Root dry weight ^1^	1.2 ± 1.6 ^a^	0.8 ± 0.6 ^a^	1.0 ± 0.9 ^a^	0.8 ± 0.5 ^a^	0.7 ± 0.7 ^a^
EM root tips	2476 ± 1545	2000 ± 1282	2988 ± 4338	2006 ± 1037	1846 ± 1701
Mycorrhization ^2^	99.6 ± 0.6 ^a^	99.0 ± 0.9 ^a^	99.0 ± 1.3 ^a^	99.6 ± 0.5 ^a^	98.2 ± 1.8 ^a^
Ergosterol ^3^	34.7 ± 9. 4 ^a^	39.5 ± 10.3 ^a^	35.6 ± 13.9 ^a^	43.4 ± 10.5 ^a^	19.0 ± 4.3 ^b^
Hyphal length	2.3 ± 1.2	3.9 ± 2.2	9.8 ± 5.5	9.9 ± 7. 8	1.6 ± 1.2

### 3.8. Ectomycorrhiza Communities of P. cembra

A total of 20 fungal OTUs were detected on *P. cembra* roots (Table 3). A mean species richness of 5.0 OTUs (SD = 1.7, *n* = 20) was detected per plot, with the highest mean richness of 6.3 OTUs (SD = 1.5, *n* = 4) detected in the N plots. There were no significant differences in richness, diversity or evenness between plots (Table 4).

**Table 3:** Potential mycorrhizal species detected on *Pinus cembra* root tips and in in-growth mesh bags (MB) buried during the vegetation period 2013 (X = detected). W = warm, K = cold, CO = control with cut vegetation, CM = untreated control, N = nitrogen fertilization.

Closest BLAST Match	Root Tips					Mesh Bags				
	W	K	CO	CM	N	W	K	CO	CM	N
*Suillus placidus*	X	X	X	X	X	-	-	-	-	-
*Rhizopogon salebrosus*	X	X	X	X	X	X	X	X	-	X
*Suillus plorans*	X	X	X	X	X	X	X	X	X	X
*Thelephora terrestris*	-	X	X	X	-	-	X	X	X	-
*Suillus sibiricus*	X	-	X	X	X	-	-	X	-	-
*Ceratobasidium* sp.	X	-	X	-	X	-	-	-	-	-
*Phialocephala fortinii* 1	-	-	X	X	X	-	-	-	-	-
*Amphinema byssoides*	-	-	X	X	-	-	-	-	X	-
*Cortinarius anomalus*	X	-	X	-	-	-	-	-	-	-
*Xerocomus ferrugineus*	X	-	-	-	-	-	-	-	-	-
*Lactarius deterrimus*	-	-	-	-	X	-	-	-	-	-
*Wilcoxina* sp.	-	-	-	X	-	-	-	-	-	-
*Articulospora tetracladia* 1	X	-	-	X	X	-	-	-	-	-
*Phialocephala fortinii* 2	-	-	-	-	X	-	-	-	-	-
*Helotiales* sp. 4	-	-	X	-	-	-	-	-	-	-
*Rhizopogon rubescens*	-	-	X	-	-	-	-	-	-	-
*Articulospora tetracladia* 2	-	X	-	-	X	-	-	-	-	-
*Helotiales* sp. 1	X	-	-	-	-	-	-	-	-	-
*Lactarius rufus*	-	-	X	-	-	-	-	-	-	-
*Meliniomyces bicolor*	-	-	-	-	-	-	-	-	-	X
*Phialocephala fortinii* 3	-	-	-	-	X	-	-	-	-	-
*Helotiales* sp. 3	-	-	-	-	-	X	-	-	-	-
*Rhizoscyphus ericae*	-	-	-	-	-	X	-	-	-	-
*Sistotrema* sp.	-	-	-	-	-	-	-	-	X	-
*Sebacinales* sp.	-	-	-	-	-	-	-	-	X	-
*Suillus* sp.	-	-	-	-	-	-	-	-	X	-
*Sebacina* sp.	-	-	-	-	-	-	-	X	-	-
*Glomeromycetes* sp.	-	-	-	-	-	-	-	-	-	X
*Phialocephala fortinii* 3	-	-	-	-	X	-	-	-	-	-

**Table 4:** Diversity indices and indicator species analysis for the mycorrhizal fungal communities associated with *P. cembra* (S = richness, E = evenness, H = Shannon’s diversity index and D’ = Simpson index) for all plots during the vegetation period 2013 (May to October).

Plot	S (*n* = 4)	E (*n* = 4)	H (*n* = 4)	D’ (*n* = 4)	Indicator Species
W	4.75 ± 1.89	0.60 ± 0.17	0.85 ± 0.37	0.45 ± 0.18	-
K	3.25 ± 0.50	0.61 ± 0.32	0.71 ± 0.34	0.41 ± 0.22	-
CO	5.50 ± 1.73	0.61 ± 0.04	1.02 ± 0.15	0.53 ± 0.04	-
CM	5.00 ± 1.41	0.56 ± 0.15	0.90 ± 0.33	0.45 ± 0.18	-
N	6.25 ± 1.50	0.70 ± 0.14	1.25 ± 0.13	0.63 ± 0.08	*S. sibiricus* (*p* < 0.03)
Season	4.95 ± 1.67	0.62 ± 0.17	0.95 ± 0.31	0.50 ± 0.16	-

**Figure 2:**
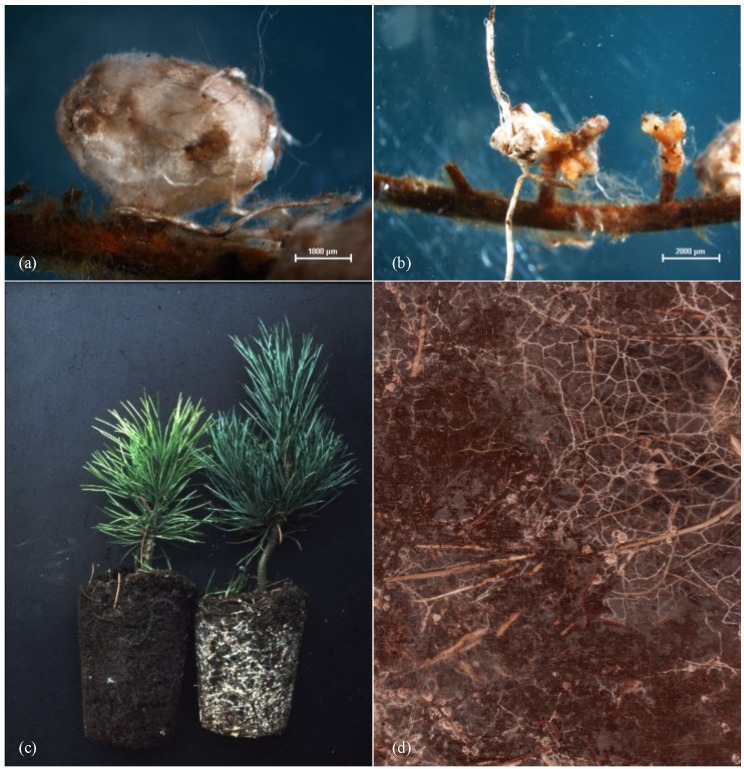
(**a**,**b**) Ectomycorrhizae of *Suillus* spp. with their nodule-like shape and emanating hyphal elements; (**c**) *P. cembra* plants as used for the afforestation in 1964, on the left without and on the right with artificial inoculation; (**d**) Rhizotron picture of *Suillus* spp. forming a large network of extraradical mycelium, with which the fungi are able to transport water and nutrients over long distances.

The three nodule-forming Suillus spp. were clearly dominating, together they had a relative abundance of 82% of the total mycorrhized root tips (Figure 2a,b). Suillus placidus was the most frequent mycorrhizal species in the afforestation with a relative abundance of 57% of the total mycorrhized root tips, followed by *S. plorans* (17%) and *S. sibiricus* (8%). Also *Rhizopogon salebrosus* was still comparatively abundant (8%). All other species had relative abundances <2%: *Rhizopogon rubescens*, *Ceratobasidium* sp. and *Thelephora terrestris* had relative abundances >1%, the other 13 mycobionts were rare. Thus, a seasonal influence was not detected for the EM communities. Species composition did not differ between treatments and/or controls.

### 3.9. The Ectomycorrhiza Inoculum Potential

A total of 13 potentially mycorrhizal OTUs were detected in the MBs, including one OTU known to form ericoid mycorrhizae (*Rhizoscyphus ericae*), and another forming arbuscular mycorrhizae (*Glomeromycota*). Only five of the remaining 11 potentially EM fungal OTUs were also identified as mycobionts on the *P. cembra* roots. Table 3 provides information on the occurrence of OTUs in the different treatments. A list of the retrieved OTUs, their respective GenBank accession numbers, and their best BLAST match are given in Table S1.

### 3.10. Effect of Soil Temperature Manipulation

Temperature manipulations and controls had a nearly identical EM species composition and diversity. The *P. cembra* roots were always almost completely mycorrhized (99%). Moreover, we detected no significant differences in SOM, pH, and SWC between the plots (Table 1).

### 3.11. Effect of Nitrogen Fertilization

We could detect a negative impact of N fertilization on the fungal biomass development and on SOM: at the end of the growing season, total C, SOM, fungal biomass and hyphal in-growth were significantly reduced in the N plots compared to the CM plots. Total N and C:N were not significantly different after fertilization. There were no significant differences in pH and SWC between fertilized and not fertilized plots (Table 1, Table 2 and Table 5). The nitrogen fertilization had no effect on the EM fungal species composition or diversity, or on the degree of mycorrhization (Table 4).

**Table 5:** Total C (%), total N (%), C:N ratio and soil organic matter (SOM; %) in control plots (CM) and fertilized plots (N) of the *P. cembra* afforestation Haggen in May and October 2013. Results are given as means (± SD). Significant differences between seasons are given at *p* < 0.05 (^1^ *p* < 0.00002; ^2^ *p* < 0.0001, ^2^ *p* < 0.03). For the effects of fertilization, values with the same suffix letter (a, b) are not significantly different at *p* < 0.05.

Plot	CM	N
*May*		
C tot ^1^	16.1 ± 3.3 ^a^	14.8 ± 1.1 ^a^
N tot ^2^	0.82 ± 0.13 ^a^	0.85 ± 0.06 ^a^
C:N	19.6 ± 1.4 ^a^	17.4 ± 1.2 ^b^
SOM	29.5 ± 4.5 ^a^	26.9 ± 2.2 ^a^
*October*		
C tot ^1^	13.4 ± 2.7 ^a^	10.5 ± 0.6 ^b^
N tot ^2^	0.70 ± 0.11 ^a^	0.64 ± 0.04 ^a^
C:N	19.1 ± 2.0 ^a^	16.3 ± 0.6 ^b^
SOM ^3^	27.2 ± 4.2 ^a^	22.1 ±1.9 ^b^

## 4. Discussion

### 4.1. The mycobionts of P. cembra in Haggen

The species richness of the *P. cembra* EM partners is relatively high in this afforestation when compared to similar *P. cembra* locations in the sub-/alpine zone [8,44], and it is within the range of the mycobiont richness as reported for other ectotrophic plants of the same altitudinal zone in the Alps: *Larix decidua* is associated with 20–30 EM species [8], *Arctostaphylos uva-ursi* with 39 [45], and *Picea abies* in Swiss forests with 28 [46].

The detected mycobiont species richness of these Swiss pines with homogenic age structure very likely represents only a part of the whole species richness of adult Swiss pines in the alpine zone: overlap with *P. cembra* mycobionts as reported from other habitats is comparatively low, but at least one of the *Suillus* specialists of *P. cembra* is always present [8,47] highlighting the importance of these specialist species for the *P. cembra* environment.

A study on the EM communities of Whitebark pine (*Pinus albicaulis*), the only stone pine occurring in North America, reported 32 EM species based on basidiomata occurrence and identification of EM from pine seedlings. Whitebark pine has declined 40%–90% throughout its range. *Cenococcum geophilum* was the most abundant and frequent EM fungus on the seedling roots, while host specialist Suilloid species were detected with low relative frequencies only [48]. *Cenococcum geophilum* was never detected in the Haggen afforestation, but it was reported from naturally established six to 10 year old *P. cembra* trees at about 100 m distance from the afforestation [20], and in the surrounding *Arctostaphylos uva-ursi* habitats [45]. Ectomycorrhizal communities from nursery-grown four to six year old *P. cembra* seedlings were dominated by *Wilcoxinia* and *Suillus* spp., but *C. geophilum* was never detected in these nurseries [8]. In a study on EM communities from four pristine *P. cembra* habitats in the South-Tyrolean Alps, *C. geophilum* was only detected on naturally rejuvenating seedlings from two sites [47]. The predominance of *C. geophilum* in the EM community of the Whitebark pine seedlings could be interpreted as an indicator of temperature stress and imply an impaired plant nutrition: *Cenococcum geophilum* is comparatively tolerant to soil heating [49], it enhances seedling water potential, but not phosphorus concentrations or photosynthesis [50].

### 4.2. The Inoculum of Ectomycorrhizae in the Soil

For colonization of new habitats, there are only few keystone spore-dispersed ectomycorrhizal fungi that can mediate pine tree invasion with help of their spore banks. *Suillus* and *Rhizopogon* spp. use a very efficient mixed colonization strategy with both rhizomorphs and spore banks being effective at colonizing pine seedling roots [51]. These Pinaceae-specific fungi are also prevalent in deer faeces, suggesting a specialized method of dispersal and potential for formation of a spore bank; thus, these fungi are uniquely adapted for dispersal and survival in treeless areas [52]. Ectomycorrhizal fungi cannot survive in deforested soils for decades or centuries without suitable hosts, or they lose their ability to form efficient EM [53]. As we know from the first unlucky afforestation attempt in Haggen, a sufficient mycorrhization with a suitable mycobiont is an absolute requirement for a successful afforestation. Dwarf shrubs such as *Arctostaphylos uva-ursi* can be a refuge for EM fungi [20,45,54], but these plants do not occur in the sampling area. When afforesting with *P. cembra* plants from nurseries, a mycobiont species richness of three to seven OTUs is usually introduced in the habitat [8]. Compared to the richness of introduced mycobionts, the richness of potentially mycorrhizal soil fungi detected in the Haggen is low: only 11 potentially EM fungal OTUs, one ericoid mycorrhizal OTU and one arbuscular mycorrhizal OTU were actively growing during the vegetation period. We therefore assume that allochthonous inoculum import is not an important factor for the establishment and survival of EM communities in this habitat, because establishment of soil EM fungi from a spore import from nearby forests (all located at lower altitudes) occurs only rarely. In contrast, fruiting of the host-specialists *S. placidus*, *S. plorans*, and *S. sibiricus* occurs regularly in high abundance in the Haggen habitat [20]. We therefore consider spore banks produced by fruiting bodies of the dominating *Suillus* spp. and *Rhizopogon* spp. as the most important inoculum present in the soil.

### 4.3. Suitability and Sustainability of Ectomycorrhiza Partners of P. cembra

Haggen is especially interesting, because this afforestation allows insight in the sustainability of mycorrhizal inoculation: information on the original inoculation of the trees in the 60s is available [53], as is a later study investigating the *P. cembra* EM partners of the afforestation in 1997 [20] allows to track mycorrhizal succession and dynamics. The original inoculation was carried out with a mixture of *S. placidus*, *S. plorans* and *S. sibiricus*, wherein especially the first two were used. In 1997, the approximately 34-year-old trees were still mycorrhized with *Suillus* species. Based on fruitbody occurrence, the species dominant in 1997 was *S. placidus* [20]. About 30 years after inoculation, *S. placidus*, *S. plorans* are still dominating, proving that inoculation with these taxa was suitable and sustainable. The suitability of inoculation with *Suillus* species could also be confirmed for other areas in Tirol and South Tyrol. *Suillus plorans*, *S. placidus* and *S. sibiricus* have always been found at different locations and at different *P. cembra* stand ages in South Tyrol [8,25,44,47]. Thus, these three *Suillus* species can be regarded as the most important and widespread symbiosis partners of *P. cembra* in the Alpine area.

### 4.4. Beneficial Properties of Suillus Host Specialists

The genus *Suillus* has >50 species, which are all host specialists with different species or genera of conifers [55]. *Suillus* spp. have co-evolved with their hosts, therefore mycobiont and photobiont are perfectly adapted to each other [56]. The three *Suillus* spp. found in the study area occur exclusively in association with *P. cembra* [57]. Their EM have a typical nodule-like shape with an extended extra-radical rhizomorph network, allowing them to quickly traverse unsuitable substrates and to efficiently transport nutrients and water: they are typical EM of the long-distance exploration type [36]. Interestingly, all mycobiont species occurring in considerable abundances in Haggen are also belonging to the long-distance exploration type (*Rhizopogon* spp., *Thelephora terrestris* and *Amphinema byssoides*). Thus, the long distance exploration type appears to be especially suitable for this undisturbed, but very stony habitat with high soil heterogeneity.

*Suillus plorans*, *S. placidus* and *S. sibiricus* are present in all age structures in the afforestation; therefore *Suillus* spp. can be regarded as typical multi-stage mycobionts. Most tree species lose unsuitable EM-partners from the nursery relatively quickly but retain suitable ones for a long time after exposure [58], but *Suillus* spp. are usually very difficult to displace [8,59]. This makes them especially suitable for a mycorrhizal inoculation.

The ectomycorrhizae of *S. tomentosus*, a host specialist of Whitebark pine, have the ability to fix nitrogen due to diazotrophic bacteria associated to the tuberculates [60]. This could be another key trait for pine invading and colonizing harsh, low fertility sites.

### 4.5. Influence of Soil Temperature Manipulation on P. cembra Ectomycorrhiza

The EM inoculation was sustainable until 2014 but the environmental conditions are changing in the future due to climate change. However, our soil temperature manipulation had no effect on the EM of *P. cembra* in this high-altitude afforestation: Temperature manipulations and controls had a nearly identical EM species composition and diversity. The *P. cembra* roots were always almost completely mycorrhized (99.35%). Moreover, we detected no significant differences in SOM, pH, and SWC between the plots. Hence, we had the opportunity to study the influence of changing soil temperatures without significant influences of side effects such as water availability or air temperatures. The CTC manipulation was successful, and no edge effects were dominant. We can therefore deduce that short-term differences in soil temperature of ±1 °C do not affect the EM communities at our study site. This is in agreement with another study carried out in a subalpine *Picea abies* forest, showing that warming of 4 °C during the snow-free season for four to five years does not affect microbial biomass nor community composition [61]. Diurnal and annual variations in alpine soils are normally very pronounced. Mean soil temperatures, maxima and minima were significantly different between our manipulated plots and controls, but the absolute maximum soil temperature of 19.5 °C (the warm plots) was apparently not negatively affecting the present fungal mycobionts. Soil temperatures within this range occur often, especially in years with high temperature extremes like e.g. in Austria in 2003 [62]. Temperature flexibility and a good adaptation potential are a prerequisite for the survival of mycobionts in such habitats. A long-lasting, sustainable mycorrhizal symbiosis like as the association of *P. cembra* with its *Suillus* host specialists can tolerate such variations, and thus persist over decades as the dominating mycobionts of *P. cembra*. However, what might happen to EM communities at elevated temperatures over the course of decades is still unknown.

### 4.6. The Effect of Nitrogen Fertilization on P. cembra Ectomycorrhiza

Nitrogen fertilization did not affect the *P. cembra* EM communities. These results are in contrast to studies observing changes in mycobiont diversity and species composition after N fertilization [63,64]. But these studies were carried out in forest environments with species-rich EM communities, which were not dominated by EM specialists. The *Suillus* EM specialists dominating in the *P. cembra* environment appear to be comparatively stress-tolerant: *in vivo*, they are not negatively affected by N fertilization within these N concentrations.

However, N fertilization caused reduced soil organic substrate availability, reduced soil fungal biomass, and reduced hyphal growth in the soil. A similar pattern was reported for a *Picea abies* forest stand in Sweden, where mycelial growth of fungi was reduced to nearly 50% compared to non-fertilized plots, and the C:N ratio dropped from 20 in the non-fertilized plots to 15 in the N fertilized plots [65]. This shows that N fertilization causes a depletion of organic material from the soil. When the amount of substrate available for the growth of soil fungi is reduced, hyphal growth and fungal biomass are also reduced.

A recent study furnishes an explanation for these observations: reduced formation rather than increased depletion of SOM. Roots and root-associated microorganisms are responsible for 50%–70% of stored carbon in boreal forest soils, because a large proportion of photosynthetically fixed C is directed belowground to roots and root-associated microorganisms [66]. Another possibility to explain the reduced SOM and fungal biomass is therefore, that the improved N nutrition of trees causes a reduction of C allocation to the root-associated microorganisms, causing a reduction of fungal extraradical growth and also of carbon sequestration to soil.

## 5. Conclusions

The mycorrhizal inoculation carried out in the 1960s *Suillus placidus*, *S. plorans* and *S. sibiricus* was so successful that temperature manipulations and nitrogen fertilization had no significant influence on species composition, diversity or mycorrhizal degree. Thus, the mycorrhization with suitable host specialists cannot be regarded only as sustainable over decades, but also as very stress-tolerant. *Suillus* species are dominating the EM community because their niche is well suited to stone pine forests at these high elevation, harsh sites. If the inoculation program had been anything else, those fungi would probably have been displaced within a few years by *Suillus*. The slow growth and wide spacing of stone pine trees at these elevations has delayed or even prevented canopy closure, further contributing to the dominance of rhizomorph- and spore-dispersed *Suillus* species. However, if these dominant keystone species would disappear in such a habitat, e.g. due to extreme changes in environmental conditions, there would be only few EM species which could replace them in their whole functional complexity. As a consequence, the whole habitat could be seriously endangered. Appropriate monitoring and management practices are recommended which guarantee the survival of suitable mycobionts in *P. cembra* habitats.

## Supplementary Files

Supplementary File 1
