# Supplementary material for: Host-Specialist Dominated Ectomycorrhizal Communities of Pinus cembra are not Affected by Temperature Manipulation
Source: J Fungi (Basel). 2015 Apr 30;1(1):55–75. doi: 10.3390/jof1010055 (PMC5770009; doi:10.3390/jof1010055)
Supplement: Supplementary File 1 [file jof-01-00055-s001.pdf]

Supplementary Materials

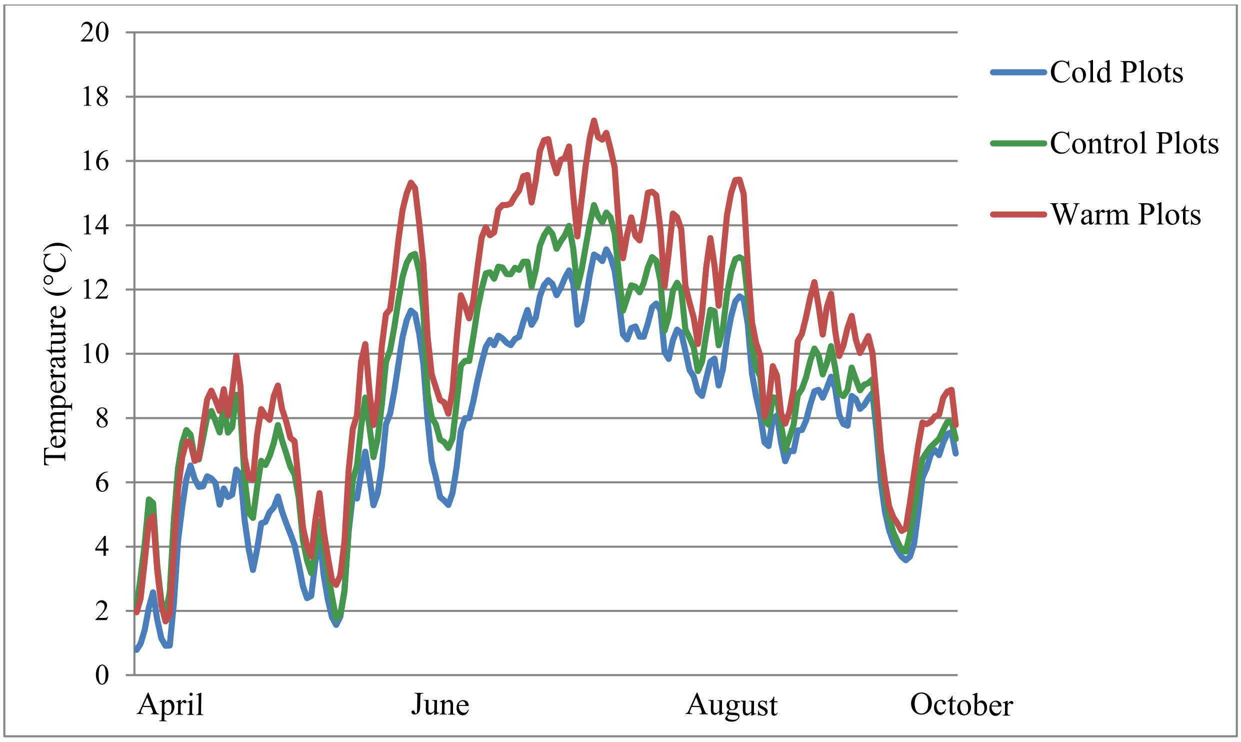

Figure S1. Soil temperatures in the manipulated plots (W, K) and in control plots (CO) during the vegetation period 2013.

Table S1. Potentially mycorrhizal OTUs detected on *Pinus cembra* root tips and/or in in-growth mesh bags (MB) buried during the vegetation period 2013. (x = detected) EM = ectomycorrhiza, AM = arbuscular mycorrhiza, DSE = dark septate endophytes.

OTU	Closest BLAST Match	Acc. Nr	GenBank Acc.Nr	EM	MB	Function
OTU8	*Amphinema byssoides*	AY838271	KM504494	x	x	EM
OTU2	*Rhizopogon salebrosus*	AY880931	KM504487	x	x	EM
OTU3	*Suillus plorans*	AJ272417	KM504489	x	x	EM
OTU5	*Suillus sibiricus*	AF166512	KM504491	x	x	EM
OTU4	*Thelephora terrestris*	HM189965	KM504490	x	x	EM
OTU13	*Articulospora tetracladia* 1	EU998918	KM504496	x	-	EM/DSE
OTU17	*Articulospora tetracladia* 2	EU998923	KM504503	x	-	EM/DSE
OTU6	*Ceratobasidium* sp.	KF646110	KM504492	x	-	EM
OTU9	*Cortinarius anomalus*	AY669645	KM504495	x	-	EM
OTU124	*Glomeromycetes* sp.	JQ272369	KM504469	-	x	AM
OTU18	*Helotiales* sp. 1	AB598090	KM504504	x	-	EM
OTU42	*Helotiales* sp. 3	KF156308	KM504511	-	x	EM
OTU15	*Helotiales* sp. 4	KF156308	KM504501	x	-	EM
OTU11	*Lactarius deterrimus*	DQ658871	KM504498	x	-	EM
OTU20	*Lactarius rufus*	KF241543	KM504505	x	-	EM
OTU21	*Meliniomyces bicolor*	HM190124	KM504506	-	x	EM
OTU7	*Phialocephala fortinii* 1	AB671499	KM504493	x	-	EM/DSE
OTU14	*Phialocephala fortinii* 2	AY394921	KM504500	x	-	EM/DSE

Table S1. *Cont.*

OTU	Closest BLAST Match	Acc. Nr	GenBank Acc.Nr	EM	MB	Function
OTU29	*Phialocephala fortinii* 3	AY524846	KM504510	x	-	EM/DSE
OTU16	*Rhizopogon rubescens*	GQ267486	KM504502	x	-	EM
OTU50	*Rhizoscyphus ericae*	AY762620	KM504414	-	x	Ericoid
OTU93	*Sebacina* sp.	DQ520096	KM504448	-	x	EM
OTU77	*Sebacinales* sp.	JQ272430	KM504437	-	x	EM
OTU76	*Sistotrema* sp.	AY805624	KM504436	-	x	EM
OTU1	*Suillus placidus*	L54118	KM504488	x	-	EM
OTU81	*Suillus* sp.	AF166512	KM504439	-	x	EM
OTU12	*Wilcoxina* sp.	HM036641	KM504499	x	-	EM
OTU10	*Xerocomus ferrugineus*	DQ066401	KM504497	x	-	EM

© 2015 by the authors; licensee MDPI, Basel, Switzerland. This article is an open access article distributed under the terms and conditions of the Creative Commons Attribution license (http://creativecommons.org/licenses/by/4.0/).
